# GoRG: Towards a GPU-Accelerated Multiview Hyperspectral Depth Estimation Tool for Medical Applications

**DOI:** 10.3390/s21124091

**Published:** 2021-06-14

**Authors:** Jaime Sancho, Pallab Sutradhar, Gonzalo Rosa, Miguel Chavarrías, Angel Perez-Nuñez, Rubén Salvador, Alfonso Lagares, Eduardo Juárez, César Sanz

**Affiliations:** 1 Research Center on Software Technologies and Multimedia Systems (CITSEM), Universidad Politécnica de Madrid (UPM), 28031 Madrid, Spain; pallab.sutradhar@upm.es (P.S.); gonzalo.rosa.olmeda@upm.es (G.R.); miguel.chavarrias@upm.es (M.C.); eduardo.juarez@upm.es (E.J.); cesar.sanz@upm.es (C.S.); 2 Instituto de Investigación Sanitaria Hospital 12 de Octubre (imas12), 28041 Madrid, Spain; apnunez@salud.madrid.org (A.P.-N.); alfonso.lagares@salud.madrid.org (A.L.); 3 CentraleSupélec, CNRS, IETR, UMR 6164, 35700 Rennes, France; ruben.salvador@centralesupelec.fr

**Keywords:** depth estimation, gpu, hyperspectral, graph cuts, multiview, medicine

## Abstract

HyperSpectral (HS) images have been successfully used for brain tumor boundary detection during resection operations. Nowadays, these classification maps coexist with other technologies such as MRI or IOUS that improve a neurosurgeon’s action, with their incorporation being a neurosurgeon’s task. The project in which this work is framed generates an unified and more accurate 3D immersive model using HS, MRI, and IOUS information. To do so, the HS images need to include 3D information and it needs to be generated in real-time operating room conditions, around a few seconds. This work presents Graph cuts Reference depth estimation in GPU (GoRG), a GPU-accelerated multiview depth estimation tool for HS images also able to process YUV images in less than 5.5 s on average. Compared to a high-quality SoA algorithm, MPEG DERS, GoRG YUV obtain quality losses of −0.93 dB, −0.6 dB, and −1.96% for WS-PSNR, IV-PSNR, and VMAF, respectively, using a video synthesis processing chain. For HS test images, GoRG obtains an average RMSE of 7.5 cm, with most of its errors in the background, needing around 850 ms to process one frame and view. These results demonstrate the feasibility of using GoRG during a tumor resection operation.

## 1. Introduction

HyperSpectral Imaging (HSI) consists of the acquisition of spatial images with information from multiple wavelength bands, starting from the UltraViolet (UV) up to the InfraRed (IR) region of the electromagnetic spectrum. This field combines classical digital imaging, where every pixel represents a small portion of a captured scene, and spectroscopy, where a limited piece of matter is characterized by its emitted energy across the electromagnetic spectrum. Accordingly, every spatial pixel of an HS image is represented by its surface reflectance across the spectrum, i.e., its spectral signature, easing its material discrimination [1].

Although HSI was initially used in remote sensing applications [2,3], its advantages boosted its utilization in different domains, such as surveillance [4], food industry [5,6], or medicine [7,8]. Concerning medicine, its benefits have been proven in several research works, as this technology is non-invasive and non-ionizing and it does not need any type of contact with the patient [9]. As a consequence, during recent years, several works [10,11,12] addressed border detection in different type of tumors, using HS images along with machine learning and image processing techniques.

In the particular case of brain cancer detection, addressed in this paper, several works benefited from using HS images in the operation room [13,14,15] to precisely detect the borders of a tumor and to help neurosurgeons during the resection process. All of these works aimed to improve both (i) the quality of the classification maps and the results of HS images, and (ii) the acceleration of the algorithms as much as possible, so the process can be executed in real time. However, these works did not consider the utilization of other data sources that are commonly used in HSI to improve classification results [16,17,18].

Currently, besides HSI, it is common that neurosurgeons use other technologies to ease the delimitation of the tumor. One of them is Magnetic Resonance Imaging (MRI) [19], which has been widely used in medicine to find anomalies within the human body. This technology is used to generate a preoperative 3D tomography (a 3D volume generated by contiguous 2D slices of a solid) of the brain, where the cancer can be distinguished. Although neurosurgeons benefit from this information, it cannot be fully entrusted, as the position of the brain changes during the trepanation; this phenomenon is known as brain shift. In order to solve this problem, IntraOperative UltraSounds (IOUS) are commonly used to match the position of the brain with the preoperative 3D tomography [20].

This work is framed in the NEMESIS-3D-CM project (http://nemesis3d.citsem.upm.es/, accessed on 10 June 2021), which aims to generate a live 3D model of the brain during a tumor resection operation using information from technologies previously introduced: HSI, MRI, and IOUS. Thus, cancer classification is expected to be more accurate than using these technologies independently. In addition, the 3D model allows for an immersive visualization of the tumor updated in real time, aiding neurosurgeons in better locating the margins of the tumor during the resection operation. The real-time constraint is therefore set to the order of a few seconds, allowing neurosurgeons to consult the model after every tissue resection on the surface of the brain.

This paper starts with the premise of generating HS 3D models in real time, addressing the task of acquiring HS images with highly detailed depth information. To this end, the authors decided to start analyzing a State-of-the-Art (SoA) technology called MPEG Depth Estimation Reference Software (DERS), able to generate high-quality depth maps from YUV images at the cost of a huge processing time. Then, this tool was GPU accelerated to prove its viability in producing high-quality depth maps in an operating room at real time. As this goal was achieved, the software was updated to use HS images as input while keeping the real time constraints fulfilled. In order to test the application, a laboratory test HS multiview sequence was employed in this work, focusing on the restrictions set by the brain cancer detection application: (i) the definition of real time to a few seconds; (ii) the target depth range, from around 0.5 m to around 1 m; and (iii) the need for a detailed depth map. In future work, the system will be tested with real brain cancer HS multiview images. Therefore, this work introduces the following contributions:

1. The analysis of a SoA tool, named MPEG (Moving Picture Expert Group) DERS, intended for immersive YUV video applications, featuring highly detailed output depth maps. This analysis is proposed to differentiate, characterize, and profile the stages included in the software.
2. The introduction and description of Graph cuts Reference depth estimation in GPU (GoRG), an accelerated YUV/HS multiview depth estimation tool based on DERS;
  (a) The GPU acceleration of one of the SoA reference implementations using global methods for depth estimation;
  (b) The development of a depth estimation tool using HS images as input, fully utilizing their information;
  (c) The GPU acceleration of all of the HS functionalities; and
3. A comparison between DERS and GoRG YUV in terms of objective and subjective quality and processing time.

The rest of the paper is structured as follows: Section 2 presents the current SoA related to HS depth estimation as well as an introduction to GPU-accelerated depth estimation algorithms. Section 3 and Section 4 include the background required to explain the proposal in this work, mainly focusing on the analysis of DERS, one of the SoA highly detailed depth estimation algorithms. Section 5 introduces GoRG, a GPU-accelerated software based on DERS able to process YUV or HS images and the proposal of this work. In Section 6, the test material and the methodology to test the proposal are explained. Section 7 includes (i) the results during the development stage of GoRG, (ii) the final results for GoRG YUV, and (iii) the final results for GoRG HS. These final results include objective and subjective qualities, processing times, and a profile of the proposal. Finally, Section 8 and Section 9 gives the final conclusions of this work along with several future lines of work.

## 2. Related Works

The idea of combining HS images with depth information has been widely studied in remote sensing, normally to improve the process of classification in any stage [18,21] (dimensionality reduction, feature extraction, classification, etc.). However, these works counted using devices that provide depth information, namely Light Detection and Ranging (LiDAR), and did not need to calculate the depth using depth estimation algorithms. Although the utilization of LiDAR may ease the computation complexity, it entails another type of problem; for example, its emitted radiation makes its use impossible in many medical applications. This is the case in the application presented in this paper.

Several recent works addressed the generation of depth information using HS images. Karaca et al. [22] introduced an HS panoramic stereo system including a rotating platform with two CCD cameras and two linescan spectrometers. Over this system, they developed a preprocessing chain and proposed a local depth estimation algorithm denominated SegmentSpectra based on multiband census transform and segment-weighted cost aggregation. It is important to notice that the authors did not focus on the acceleration, so although it is a local stereo algorithm, the processing time ranges around more than a hundred seconds. Another example is the work of Heide et al. [23], who presented an HS stereo system with two snapshot cameras and a local processing chain based on a weighted sum of census transform, Sum of Absolute Differences (SAD), and Sum Gradient Differences (SGD), along with a guided filtering process. In addition, their work focused on the acceleration of the application in a GPU, where they obtained results in the order of hundred of milliseconds.

To the best of the authors’ knowledge, strategies to correctly extract the depth from the multiview HS information are only addressed in previous works. As explained before, these works aim to provide strategies using local methods to ease the computational requirements that process HS. In addition, the application of these works focuses on object recognition, where the level of quality detail required is not as high as in applications of immersive vision, which is the quality level desired in this work.

In the YUV/RGB domain, however, the SoA contains many different methods to obtain a depth estimation from two or more cameras. For HS images, many local methods were proposed during recent years [24,25,26] aiming to produce results rapidly without compromising the quality of the depth maps, which normally are noisy or their borders are not well defined. Some of them were also tested on GPU accelerators, obtaining processing times at around tens of milliseconds [27,28]. In this domain, global methods or semi-global methods were also employed to improve the quality of results at the cost of spending more time processing. One example is the work of Chai et al. [29], with a semi-global stereo matching algorithm based on minimum spanning tree that achieves better quality results than other SoA algorithms except for graph cuts [30]. Although their algorithm is less complex than graph cuts, they did not include a processing time comparison.

Considered the algorithm with the best quality depth map generation, graph cuts were implemented in DERS to fulfill the quality requirements of immersive video applications. The quality and processing time needed by this software were studied in another work [31], showing that for generating these highly detailed depth maps, the processing time needed oscillates between hundred and thousand of seconds. Therefore, although DERS can be considered a higher bound in depth map quality, its high processing time forces its use offline.

To conclude this section, it is important to remark that, within the medicine field, although HS images are common, the use of depth information is rare. The SoA in this context is scarcely the work introduced by Randeberg et al. [32], where they used HS images and depth information to better characterize non-healing skin ulcers. In their work, however, the depth information is not generated from the HS cameras but obtained from an YUV camera stereo setup.

## 3. Background: MPEG Depth Estimation Reference Software (DERS)

**Figure 1:**
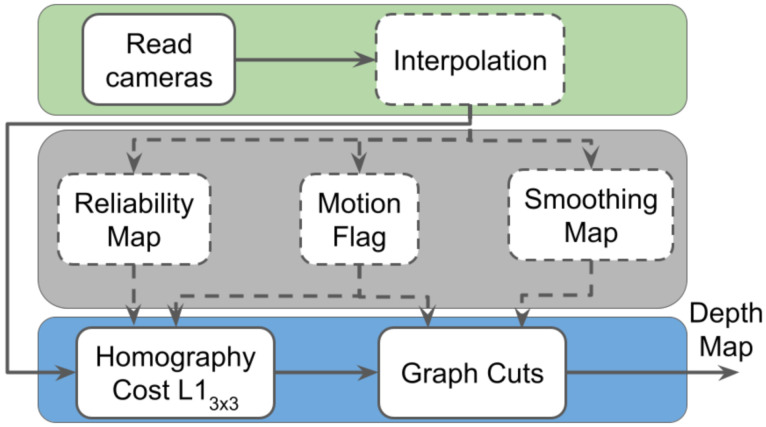
DERS structure.

MPEG DERS [31] has been the reference software for depth estimation in MPEG-I for several years. Its use is mainly focused on the generation of high-quality depth maps for immersive video applications using YUV images as input without considering the processing time needed. For this reason, the algorithm chain relies on a computationally intensive global optimizer, which improves the quality of the depth maps by smoothing similar color areas while preserving borders. Its main stages are depicted in Figure 1, which are analyzed in detail in the next subsections. In the figure, a green background represents initialization, a gray background represents auxiliary processes, and a blue background represents core processes. The dashed line means optional.

### 3.1. Read Cameras

In this stage, all of the information from the YUV cameras is introduced in the system. It includes the colored image from the central camera view and for the neighbor cameras, which are normally positioned above, below, on the left, and on the right. This information also comprises the intrinsic and extrinsic parameters, which are needed to know how the light is projected within the camera and where the camera is located in the real world, respectively.

### 3.2. Homography Cost

**Figure 2:**
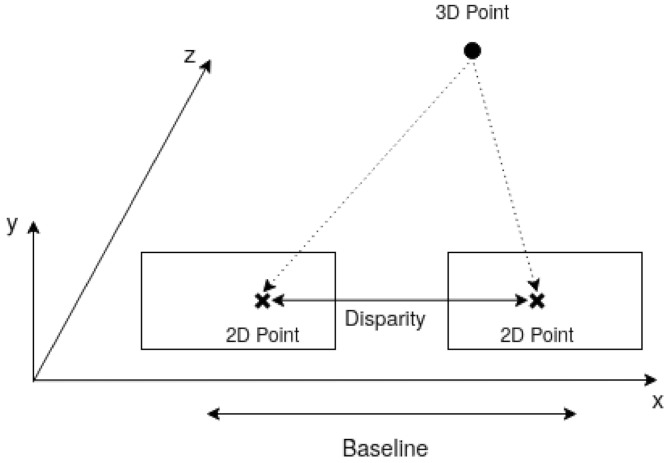
Two-camera setup for depth estimation.

This is the main stage in DERS, as it is where the geometrical information of the cameras is used to obtain the depth map. It is based on the principle that the distance from a camera to a 3D point, i.e., the depth of a pixel, depends on the distance between the 3D point projected in cameras located in different positions. The simplest setup, in Figure 2, consists of two cameras located in the same plane and separated a certain distance, denoted as baseline. If the distance between the 2D projection in both cameras, disparity, is known, then simply solving the triangle with trigonometry rules finds the distance from the cameras plane to the 3D point.

The difficult part in the previous process is not the calculation but finding the 2D projection of the same 3D point on different cameras, and therefore, all efforts are put in finding this correspondence. In addition, real setups can be much more complex than two co-planar cameras, hence needing an algorithm able to extract the depth information for cameras located in any position and with any orientation.

**Figure 3:**
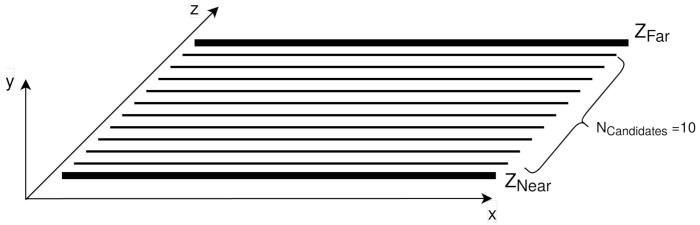
Sweeping plane parameters.

To achieve this goal, in DERS, the sweeping plane algorithm is employed. This algorithm needs three basic parameter inputs from the user (along with the intrinsic and extrinsic parameters of the cameras in the setup): $Z_{near}$, $Z_{far}$, and $N_{candidates}$. These parameters are the nearest and farthest distances where the scene is placed and the depth resolution of the estimation, i.e., the number of intermediate planes between $Z_{near}$ and $Z_{far}$. This is defined to limit the problem to a certain portion of the space and with a non-infinite resolution. In this way, the pixel depth estimated is always included in one of these planes. This idea is depicted in Figure 3.

**Figure 4:**
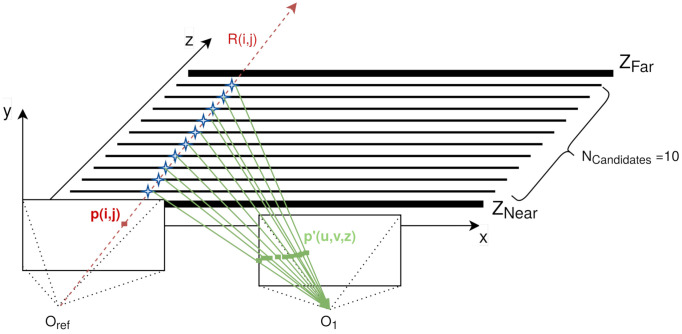
Example of the candidate finding process for pixel *p*(*i*,*j*).

With the limited 3D space defined and starting from the central camera, from now on, in the denominated reference camera, all of its pixels follow the following process (as depicted in Figure 4):

1. An infinite ray R(i,j) between the reference camera optical $O_{ref}$ axis and the pixel p(i,j) is created using the intrinsic and extrinsic parameters of the reference camera (in red in Figure 4).
2. This ray R(i,j) is intersected by the intermediate planes between $Z_{near}$ and $Z_{far}$, leading to $N_{candidates}$ 3D points (x,y,z), each with known *z*. From now on, these *z* values are called $Z_{candidates}$, as one of them is the final depth (in blue in Figure 4).
3. These 3D points are projected in another camera as in step 1, using the intrinsic and extrinsic parameters of that camera (in green in Figure 4). These 2D points $p^{\prime }\left(u,v,z\right)$ in the new camera are denominated *candidates*, and every one of them has a $Z_{candidate}$ associated.

This process is called homography transformation, and it is formulated as follows:

(1) $$\frac{1}{s}\left[\begin{array}{c} u\\ v\\ s\\ 1 \end{array}\right]=\left(\frac{K_{cam}\left[R_{cam}|t_{cam}\right]{\left(\frac{K_{ref}\left[R_{ref}|t_{ref}\right]}{0\;\;0\;\;0\;\;1\;}\right)}^{-1}}{0\;\;\;\;0\;\;\;\;0\;\;\;\;1}\right)\left(\begin{array}{c} i\\ j\\ 1\\ 1/z \end{array}\right)$$

where (from right to left) (i,j) is the pixel index from the reference camera, *z* is the value of a $Z_{candidate}$, *K* is the intrinsic matrix of a camera, *R* is the rotation matrix of a camera, *t* is the translation vector of a camera, (u,v) is the pixel index in the neighbor camera, and *s* is an undetermined constant needed to correctly scale the 2D point. Once the value is obtained, it is rounded and asserted that *x* is between 0 and the width of the camera image and that *y* is between 0 and the height of the camera image. In this way, it is ensured that the candidate has a corresponding pixel in this neighbor camera (if not, that is not a valid candidate).

**Figure 5:**
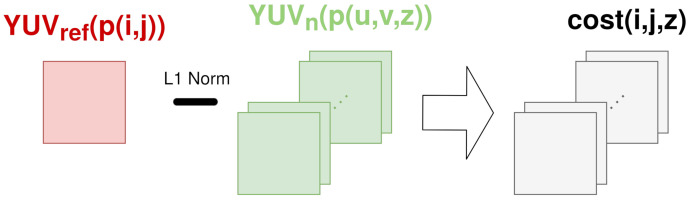
Example of cost for pixel *p*(*i*,*j*) using a 3×3 window.

The last step is the generation of a cost for every candidate (that in turn is a cost for a $Z_{candidate}$) in a reference pixel. This cost indicates the likelihood of a reference pixel having a certain $Z_{candidate}$ as the depth (more likely if it is smaller). To do so, all of the corresponding pixels in the neighbor camera are compared to the reference pixel using a weighted 3×3 L1 norm in Y (prioritizing central and cross pixels) plus a singular L1 norm in U and V. This is depicted in Figure 5. If this process is repeated for every pixel and $Z_{candidate}$ for a pair of cameras, the result is an initial cost cube.

The described process is used to obtain an initial cost cube for a pair of cameras. To obtain a cost cube that considers an uncertain number of cameras, in DERS, the sweeping plane is repeated for every pair of cameras (between the reference and all the neighbors) and, then, the minimum cost for pixel and $Z_{Candidate}$ is chosen. In this way, the global minimum cost for all of the cameras is selected. Additionally, the contribution of a neighbor camera is slightly weighted depending on its distance to the reference camera (if the camera is far, the weight is higher, meaning that it is more likely that the minimum cost is found in a near camera).

### 3.3. Graph Cuts

Graph cuts is a global energy minimization algorithm implemented in different applications, including depth estimation [30] that uses a graph structure to define and solve the minimization problem. In DERS, it is used to obtain a global optimized depth map from the initial cost cube generated in the homography stage.

As stated in Section 3.2, in the initial cost cube, every pixel of the reference camera has an associated cost for every $Z_{candidate}$, where a smaller costs mean that it is more likely that this pixel has $Z_{candidate}$ as the depth. Hence, the simpler approach to finding a depth map would be to calculate the minimum cost within a pixel and to assign $Z_{candidate}$ as the depth.

However, using graph cuts, not only the cost is considered to find the final depth map. The energy function that minimizes this algorithm considers not only the cost of assigning a pixel to a certain depth but also if neighbor pixels have similar depths, guaranteeing depth continuity. This energy function is formulated as follows:

(2) $$E\left(f\right)=E_{data}\left(f\right)+E_{smooth}\left(f\right)=\sum_{p\in P}R\left(p\right)D\left(p,f_{p}\right)+\sum_{(p,q)\in N}S_{p,q}\left(p,q\right)V_{p,q}\left(f_{p},f_{q}\right)$$

where *f* is a label that generates a certain energy, a depth map in this case, and where P are the image pixels and N is a neighborhood of pixels. The data term $R\left(p\right)D\left(p,f_{p}\right)$ measures the cost of assigning a pixel to a certain depth, based on the homography information. R(p) refers to the reliability map (further explained in Section 3.5), which is used to scale the cost. The smoothing term, $S\left(p,q\right)V_{p,q}\left(f_{p},f_{q}\right)$, measures the cost of associating a depth to a pixel, taking into account its neighbors’ depths and similarity; for this reason, this term also includes the smoothing map (further explained in Section 3.6). The use of this map ensures that similar neighbors, based on their color in the reference camera, have similar depths.

**Figure 6:**
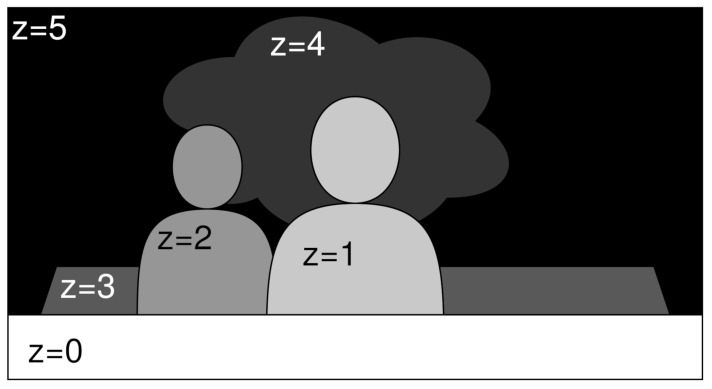
Alpha expansion example. The gray tones indicates depth levels, where black is the farthest and white is the nearest.

To find the labelling, i.e., a depth map that minimizes the energy of this function, DERS relies on the Kolmogorov library [30] using the alpha expansion method. This strategy consists of dividing the three-dimensional starting graph, the initial cost cube, into multiple 2D graphs, one for the plane in the initial cost cube. In this way, the label is updated iteratively, adding the depth to the depth map plane by plane. This is depicted in Figure 6, where *z* is an example of $Z_{candidate}$ and the gray level indicates proximity, from white (nearest) to black (farthest).

### 3.4. Interpolation

This stage is intended to improve the results of the depth estimation by generating larger input images. The underlying idea is that, in the homography stage (refer to Section 3.2), pixels from the central camera are projected in other cameras, and therefore, their geometrical positions cannot exactly fit in the geometrical position of the pixels in those cameras. To solve this problem, these geometrical positions are rounded to the nearest pixel. However, during this rounding process, some information is lost.

**Figure 7:**
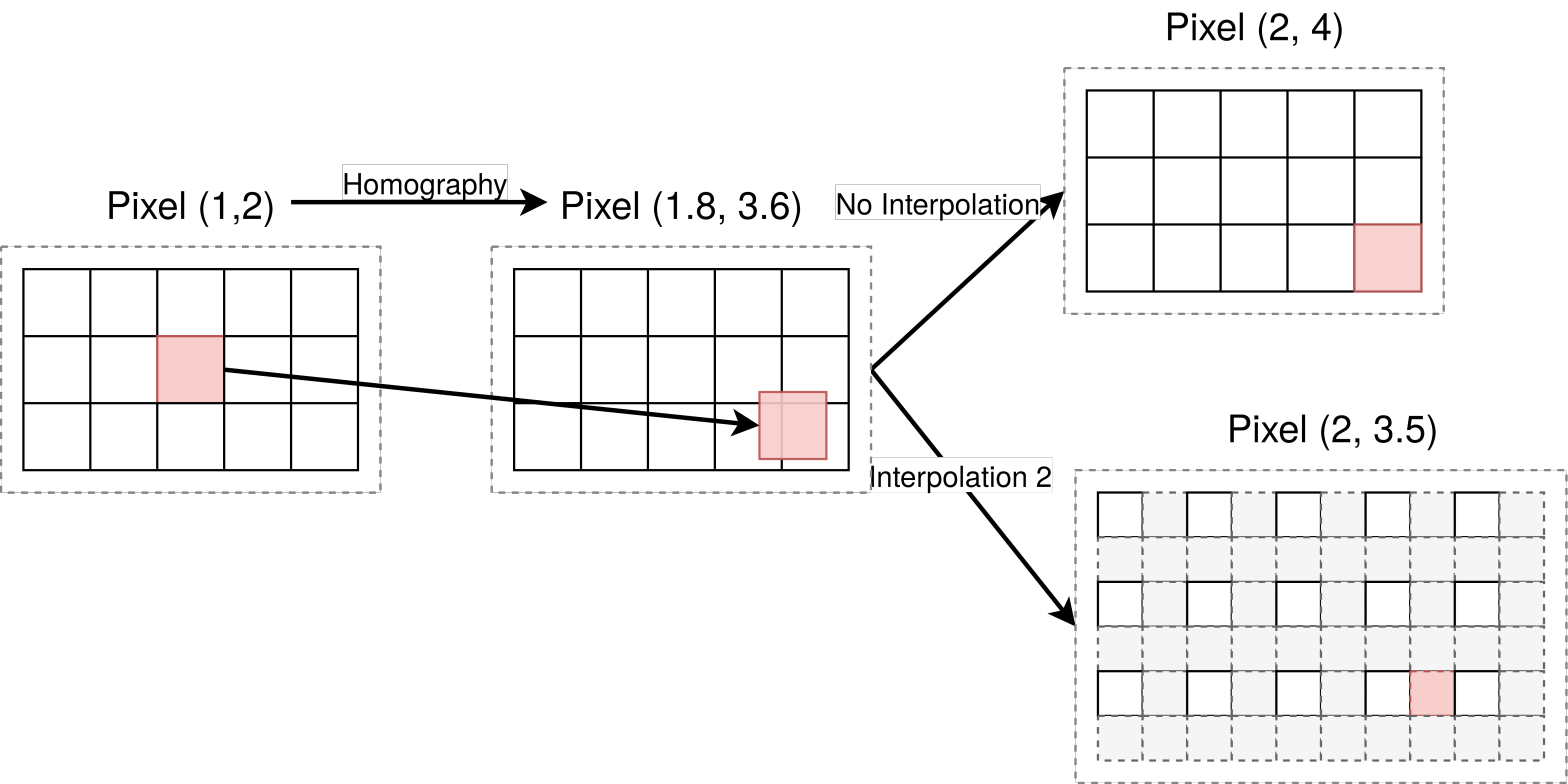
Interpolation example.

This is represented in Figure 7 with an example where pixel (1, 2) is projected to pixel (1.8, 3.6) onto another camera. If no interpolation is performed, the projected pixel is considered pixel (2, 4). For a factor-two interpolation, the projected pixel is pixel (2, 3.5). This changes the initial cost cube from the homography stage, as the intensities in pixel (2, 4) are not necessarily the same as in pixel (2, 3.5). The interpolation in DERS is changeable, and it is possible to change its factor and its type: nearest neighbor, linear, or cubic interpolations.

### 3.5. Reliability Map

The reliability map is an optional stage that produces a reliability image based on the central camera and depends on the level of texture in a pixel region, which means the level of color differences in a neighbor window. This information is used in the homography step to enlarge the costs where the texture is low, as they are less reliable than the high textured ones.

**Figure 8:**
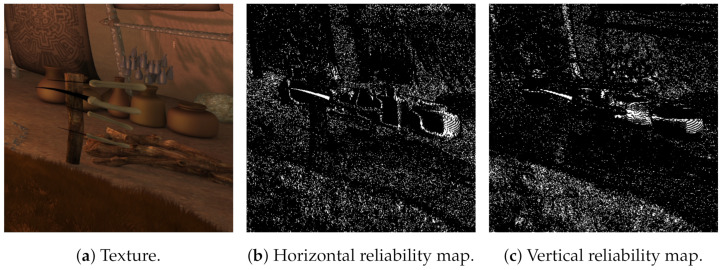
Detail of a sequence. For reliability maps, whiter values mean less reliability. Original sequence Orange Shaman, reproduced with permission from G. Clare (Orange); Orange Shaman is a derivative work of the Blender Durian project under “Creative Commons Attribution 3.0” license.

A reliability map is calculated as the average horizontal and vertical differences for every pixel in a 3×3 neighbor window and limited by a user-defined threshold [31]. Some details are depicted in Figure 8 for the textured image and the horizontal and vertical reliability maps with a threshold of 10 (average differences higher than 10 are limited to 1, lower differences are mapped to values between 0 and 1).

### 3.6. Smoothing Map

The smoothing map is an optional stage that generates an image in which every pixel indicates the smoothness level in the central camera for a neighbor window. It is used in graph cuts to scale the connection between nodes, meaning that, if the nodes have similar color, they are more strongly connected and hence more likely belonging to a similar depth.

**Figure 9:**
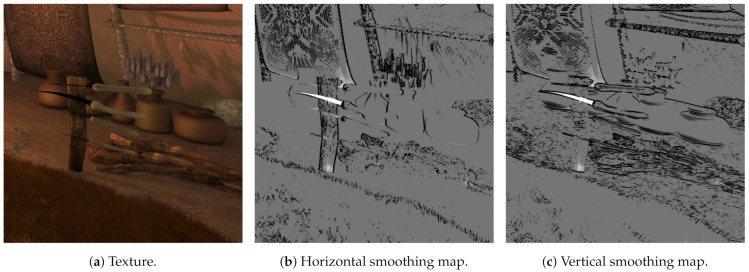
Detail of a sequence. For smoothing maps, whiter values mean smoother. Original sequence Orange Shaman, reproduced with permission from G. Clare (Orange); Orange Shaman is a derivative work of the Blender Durian project under “Creative Commons Attribution 3.0” license.

Smoothing map is calculated as the average horizontal and vertical differences for every pixel in a 3×3 neighbor window, limited and scaled by two user-defined parameters [31]. Some details are depicted in Figure 9 for the textured image and the horizontal and vertical smoothing maps with a threshold of 24 and a scaling constant of 0.2.

### 3.7. Motion Flag

The motion flag is a boolean image that represents the differences between the current frame and the previous one. It is used both in the homography and graph cuts stages to ensure temporal consistency and to speed-up the process, as the parts that did not change are not updated. It is calculated with an average difference user-parametrizable window that compares, using the L1 norm, the current frame with the previous one; if the value obtained is greater than a threshold, which is another parameter, the pixel is considered changed.

## 4. Background: CUDA Cuts

CUDA Cuts [33] is a GPU-accelerated graph cuts considered one of the most important GPU implementations in the SoA. Based on the push-relabel algorithm, this parallel implementation makes use of shared memory to solve up to 60 graph cuts per second for 1024×1024 pixel images (15 ms period). These results, compared to the Kolmogorov library (the one used in DERS), are between 40 and 60 times faster, although for obtaining this acceleration, some losses are introduced [34]. For this reason, one implementation of CUDA Cuts (https://github.com/metthueshoo/graphCut, accessed on 10 June 2021) was studied and incorporated into the proposal of this work. This implementation is intended for the segmentation of an image to a binary image (foreground and background) by creating a graph with a certain weight for every pixel and connections with its neighbors. However, as explained in Section 3.3, this process needs to be inserted into the alpha expansion loop in order to obtain the final depth map from the initial cube cost. The process of adapting this implementation to the alpha expansion loop within GoRG is explained in Section 5.3.

## 5. Proposal: Graph Cuts Reference Depth Estimation in GPU (GoRG)

Graph cuts Reference depth estimation in GPU (GoRG) is a GPU-accelerated application intended to obtain depth maps either from YUV or HS images. This application was conceived as an accelerated software based on DERS able to obtain similar results in terms of quality using a video synthesis processing chain while obtaining processing times under tens of seconds. For this reason and prioritizing the acceleration, GoRG includes two functional differences to DERS that produce quality losses: (i) interpolation is not included in GoRG, as the quality-time trade-off is not considered beneficial (further explained in Section 5.4) and (ii) CUDA Cuts library introduces appreciable quality losses at the cost of accelerating graph cuts algorithm (see Section 4).

In the following sections, the YUV acceleration is explained, as a prior step to understand the HS-accelerated version, which also includes the strategies followed to employ HS images as input. The results of this implementation are exposed and discussed in Section 7.

### 5.1. Read Cameras

In this stage, the central image along with the four nearest images from the multiview dataset are incorporated into the application, either they are YUV or HS, and are able to work in these two different modes. To avoid unnecessary data movements, these images are transferred to the GPU memory at this point, as all of the following computations do not make use of any other large CPU data. In this way, only two CPU–GPU data transactions are performed per frame: the input images and the retrieval of the final depth map. Intrinsic and extrinsic camera parameters are read as well.

### 5.2. Homography Cost

**Figure 10:**
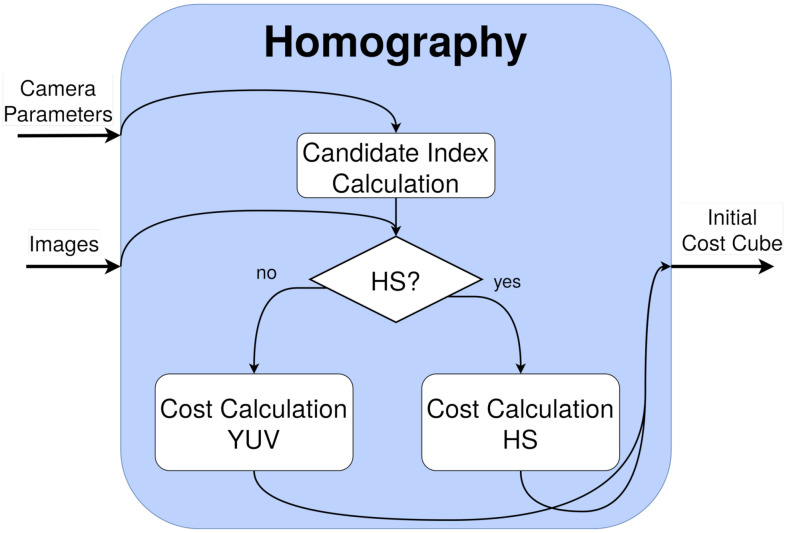
Kernels employed in the homography cost stage.

This stage is divided into two different kernels (parallel functions in GPU) to improve its acceleration. The scheme is depicted in Figure 10. These kernels are further analyzed in Section 7.1.1.

The first kernel, the candidate index calculation, calculates the indexes for all of the depth candidates for every pixel. This process is completely independent for every pixel and candidate, and only a matrix–vector multiplication (Equation (1)), scale, and rounding is required per element, as seen in Algorithm 1. This is implemented through a kernel that addresses a thread per pixel and iterates for the number of depth candidates (instead of a number of threads equal to the total number of independent elements).

The second kernel is the cost calculation using the previous indexes and is different depending on the image nature. They are introduced in Algorithm 2 and Algorithm 3, respectively:

1. For YUV images, this kernel compares the reference camera image to one secondary camera, employing the corresponding candidates, by means of a L1 3×3 neighbor weighted window in Y and a pixel L1 norm for U and V (exactly as in DERS, see Section 3.2). As in the previous kernel, every thread is addressed to a pixel and iterates through the depth candidates.
2. For HS images, this kernel employs a 3×3 neighbor weighted window, with a different cost function: SID-SAM [35]. This function is the combination of two well-known HS measures: Spectral Information Divergence (SID) and Spectral Angle Mapper (SAM), widely used to compare HS signatures and usually, to classify HS pixels. Its equation can be seen in (3), where *p* and *q* are two HS pixels and *B* is the number of bands. The thread distribution is similar to the previous one, with the difference of a new loop inside every thread intended to read and accumulate the spectral values of an HS pixel (located within SIDSAM function in Algorithm 3).

**Algorithm 1** Candidate index calculation kernel.1:**for** $pixel_{x,y}$ in parallel **do**2:    **for** $z=1,\ldots ,N_{candidates}$ **do**3:        $pixel_{x^{\prime },y^{\prime },z^{\prime }}^{\prime }=H\times {\left[x,y,z\right]}^{T}$4:        $pixel_{x^{\prime },y^{\prime },z^{\prime }}^{\prime }=round\left(pixel_{x^{\prime },y^{\prime },z^{\prime }}^{\prime }/pixel_{z^{\prime }}^{\prime }\right)$5:    **end for**6:**end for**

**Algorithm 2** Cost candidate YUV calculation kernel.
1:**for** $pixel_{x,y}$ in parallel **do**2:    Read and store in registers 3x3 window $Y1_{x,y}$3:    **for** $z=1,\ldots ,N_{candidates}$ **do**4:        $cost_{x,y,z}=\sum_{window}L1\left(Y1_{x,y},Y2_{x^{\prime },y^{\prime },z}\right)$5:        $cost_{x,y,z}+=L1\left(U1_{x,y},U2_{x^{\prime },y^{\prime },z}\right)$6:        $cost_{x,y,z}+=L1\left(V1_{x,y},V2_{x^{\prime },y^{\prime },z}\right)$7:    **end for**8:
**end for**



(3) $$\begin{array}{c} SID\left(p,q\right)=\sum_{b=1}^{B}p_{b}log\left(p_{b}/q_{b}\right)+\sum_{b=1}^{B}q_{b}log\left(q_{b}/p_{b}\right)\\ SAM\left(p,q\right)=cos^{-1}\left(\frac{\sum_{b=1}^{B}p_{b}q_{b}}{\sqrt{\sum_{b=1}^{B}p_{b}}\sqrt{\sum_{b=1}^{B}q_{b}}}\right)\\ SIDSAM(p,q)=SID(p,q)\times tan(SAM(p,q)) \end{array}$$

**Algorithm 3** Cost candidate HS calculation kernel.
1:**for** $pixel_{x,y}$ in parallel **do**2:    **for** $z=1,\ldots ,N_{candidates}$ **do**3:        $cost_{x,y,z}=\sum_{window}SIDSAM\left(HS1_{x,y},HS2_{x^{\prime },y^{\prime },z}\right)$4:    **end for**5:
**end for**



### 5.3. Graph Cuts

This stage is developed on top of the CUDA Cuts GPU implementation (see Section 4), a library that accelerates the process of segmenting a given graph to a binary image (foreground and background). In GoRG, it is employed within the alpha expansion method to calculate which pixels belong to the current considered depth. To achieve this goal and to avoid unnecessary CPU–GPU memory movements, a rework of CUDA cuts library implementation was performed that significantly improves CPU–GPU data transactions. The main changes and additions to the API are described below:

1. The library was adjusted to feature a full GPU interface, where both the input graph and the output segmentation are in the GPU, respectively.
2. Every memory allocation, de-allocation, or initialization process was implemented in separate functions, allowing its use before or after the alpha expansion process and hence avoiding repeating them every iteration.
3. Two kernels were developed to directly manage inputs and outputs from the library. The first kernel initializes the graph weights using information from the homography and the second kernel extracts the results after the graph cut to update the final depth map.

**Figure 11:**
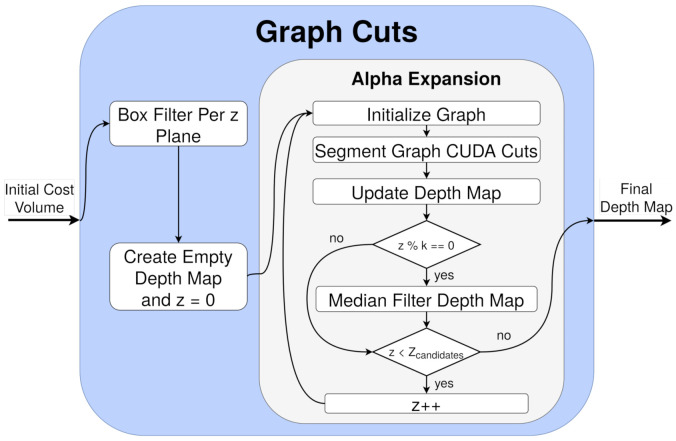
Cuda Cuts scheme in GoRG.

In addition, CUDA Cuts may yield suboptimal results [34], which produces slight errors, specially in borders and small details. As the alpha expansion method updates the depth map based on the previous calculation of graph cuts, this error is accumulated plane by plane, impacting largely the resulting quality. As a consequence, two filters were implemented to improve the quality of the results: (i) a l×l box filtering plane by plane in the initial cost volume and (ii) a n×n median filtering in the final depth map every *k* iterations. The scheme of this process can be seen in Figure 11, and the evaluation of the filtering is detailed in terms of quality and processing time in Section 7.1.2.

This process is applied to the initial cube of costs, created either from YUV or HS images; for this reason, there is not a special treatment for HS images. The only difference is the values of *n* and *k*: for YUV images, n=7 and k=30, while for HS images, n=3 and k=30. These changes are mainly due to the size of the YUV and HS images tested. For both cases, l=5.

### 5.4. Interpolation

The interpolation in GoRG is not implemented given the trade-off results obtained for DERS between no interpolation or 2, 4, and 8 factor interpolations [36] (tested for YUV). This evaluation is analyzed and discussed in Section 7.1.3.

### 5.5. Reliability and Smoothing Map

These two auxiliary processes are jointly accelerated for horizontal and vertical maps, hence needing two kernels: one for the vertical reliability and smoothing map and the other for its horizontal counterpart. They are different for YUV and HS images and aree xplained below. The vertical map kernels for YUV and HS are introduced in Algorithms 4 and 5, respectively.

1. For YUV images, the process is implemented through a kernel that addresses a pixel per thread. Every thread accumulates the difference between the left three-pixel-column and the right three-pixel-column to the central pixel in the horizontal map kernel and the difference between the down three-pixel-row and the up three-pixel-row to the central pixel in the vertical map. Then, the difference is processed using the corresponding user-defined parameters (see Section 3.5 and Section 3.6).
2. For HS images, kernels are similar, implementing a L1 norm averaged by bands as cost function and hence notably increasing the number of data load required.

**Algorithm 4** Vertical YUV smoothing/reliability map kernel.1:**for** pixel in parallel **do**2:    $gap_{x,y}=\sum_{\hat{y}=-1}^{1}Y_{x-1,\hat{y}}-Y_{x+1,\hat{y}}$3:    $gap_{x,y}=abs\left(gap_{x,y}\right)$4:    $gap_{x,y}+=U_{x-1,y}-U_{x+1,y}$5:    $gap_{x,y}+=V_{x-1,y}-V_{x+1,y}$6:    $gap_{x,y}=abs\left(gap_{x,y}\right)$7: 8:    $relV_{x,y}=f\left(gap_{x,y}\right)$9:    $smthV_{x,y}=f\left(gap_{x,y}\right)$10:**end for**
**Algorithm 5** Vertical HS smoothing/reliability map kernel.1:**for** pixel in parallel **do**2:    **for** b=1,…,bands **do**3:        $gap_{x,y}+=\sum_{\hat{y}=-1}^{1}HS_{x-1,\hat{y},b}-HS_{x+1,\hat{y},b}$4:    **end for**5:    $gap_{x,y}=abs\left(gap_{x,y}\right)$6: 7:    $relV_{x,y}=f\left(gap_{x,y}\right)$8:    $smthV_{x,y}=f\left(gap_{x,y}\right)$9:**end for**

### 5.6. Motion Flag

This process is accelerated in GoRG only for YUV images due to the lack of multiview HS video sequences to test. Future work will address the development of this stage by taking into account HS features changing from one frame to the following.

For YUV images, this process is accelerated into a single kernel that compares, for each pixel in a thread, if an user-defined window changes from the last processed frame more than a user-defined threshold. It is depicted in Algorithm 6.
**Algorithm 6** Motion map YUV kernel.1:**for** pixel in parallel **do**2:    $diff_{x,y}=\sum_{window}L1\left(Y1_{x,y}^{prev}-Y1_{x,y}\right)$3:    $motion_{x,y}=\left(diff_{x,y}>threshold\right)$4:**end for**

## 6. Experiment Setup

In this section, the experiment setup is stated, giving details about the material and methods employed to test GoRG. It includes YUV and HS material, as testing GoRG with YUV images was a crucial step in analyzing and developing its HS development.

### 6.1. YUV Material

For testing GoRG in YUV, a set of eight multiview sequences from MPEG-I is used [37,38]. Their identifiers and features are introduced in Table 1. Quality and processing time results are compared to DERS in Section 7.2.

**Figure 12:**
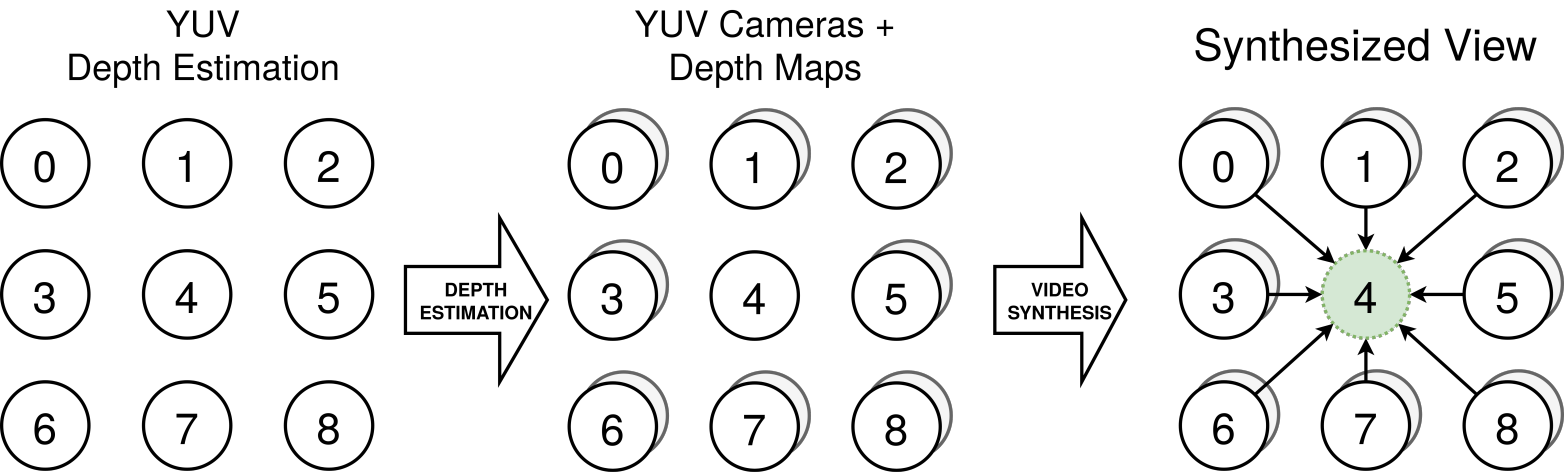
Processing chain employed to test YUV images.

These sequences do not count using depth map ground truths, hindering its quality assessment. For these reason and taking into account that they are intended for immersive video applications, their quality is evaluated through a complete immersive video chain. In this chain, the multiview dataset is used (i) to obtain all of the depthmaps associated with every real camera and (ii) to synthesize, using a video synthesizer, a virtual camera in the same position of the real cameras, using information of depth and color from its surrounding real cameras. This is depicted in Figure 12, illustrating the process for camera 4. After this chain, a synthesized image, completely dependent on the depth maps, is generated in exactly the same position as a real camera. This can be compared pixel by pixel to the real camera image, obtaining an objective metric for quality assessment.

In this work, the image synthesizer software employed is Versatile View Synthesizer (http://mpegx.int-evry.fr/software/MPEG/Explorations/6DoF/VVS, accessed on 10 June 2021) [39], and the metrics employed for objective quality are Weighted Spherical PSNR (WS-PSNR) [40], Immersive Video PSNR (IV-PSNR) [41], and Video Multimethod Assessment Fusion (VMAF) [42].

### 6.2. HS Material

For testing GoRG with HS images, a dataset of multiview HS images emulating operating room conditions was obtained using the NEMESIS-3D-CM acquisition system. These results are introduced in Section 7.3.

**Figure 13:**
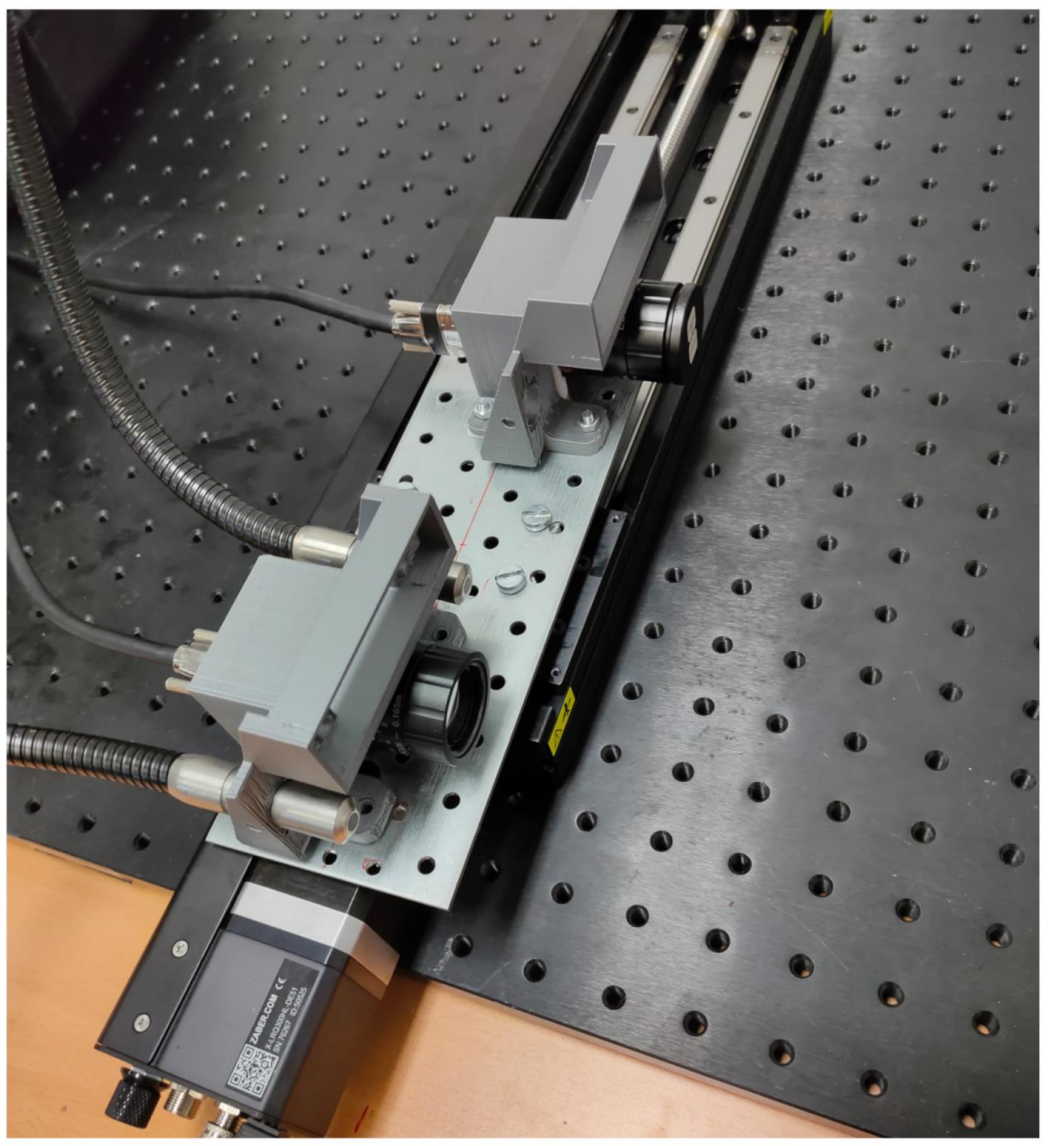
Laboratory test acquisition system.

This system comprises two different HS cameras mounted on a precise linear actuator. The laboratory testing setup is depicted in Figure 13.

1. The first HS camera is a snapshot camera, as it features a squared filter pattern with 5×5 spectral bands that allows for the acquisition of HS images in videos with up to 170 Frames Per Second (FPS) without the need for moving either the camera or the objects. The acquisition of these spectral bands implies a reduction in the spatial resolution, as five correlative pixels in each dimension (squares of 5×5) are considered the same spatial pixel in different spectral bands. For this reason, this camera is able to acquire 409×217×25 (width×height×bands) images in a video streaming fashion.
2. The second camera is a linescan camera, with a row filter pattern and150 spectral bands. This camera needs precise movement through rows, so that the captured object is completely acquired for every spectral band. After the scanning process, images of 2045×1085×125 (width×height×bands) are generated.

This system uses the movement necessary for obtaining high-resolution (both spatial and spectral) HS images with the linescan camera to obtain multiview low-resolution HS images as well. Therefore, only the snapshot HS images are used in this work.

**Figure 14:**
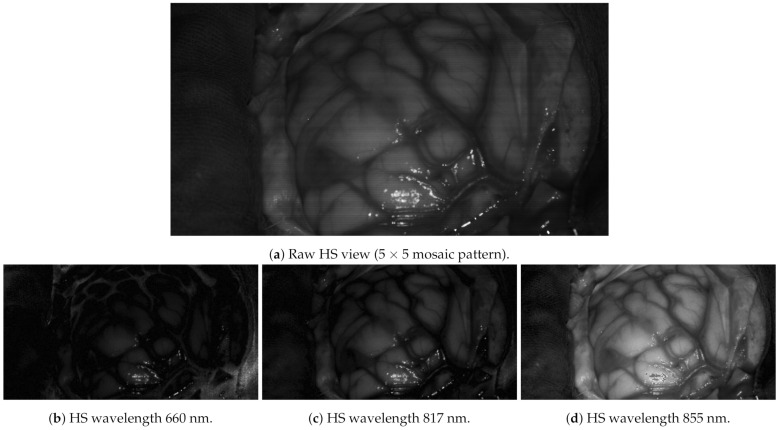
Brain cancer operation HS image example. Only three of its 25 bands are represented.

The multiview dataset was acquired in a laboratory using the same illumination conditions as the operating room and preprocessed to test GoRG. Preprocessing comprises radiometric calibration, cube generation, spectral correction, and interpolation at a factor of 2, leading to 832×448×25 images. The dataset information is depicted in Table 2. It is important to notice the reduced depth range and depth steps, 60 cm and 100 steps, respectively; compared to the YUV sequences, this range is limited. This is designed to emulate functioning in the operating room. As an example, Figure 14 shows a real HS image captured during a surgical operation.

**Figure 15:**
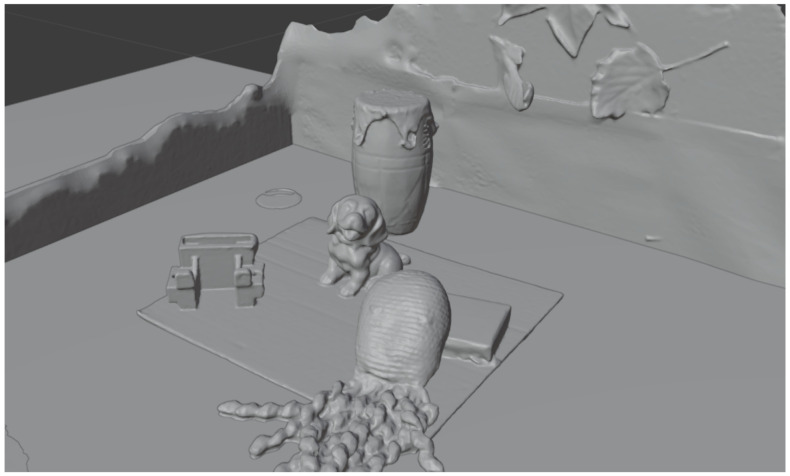
Three-dimensional model ground truth.

**Figure 16:**
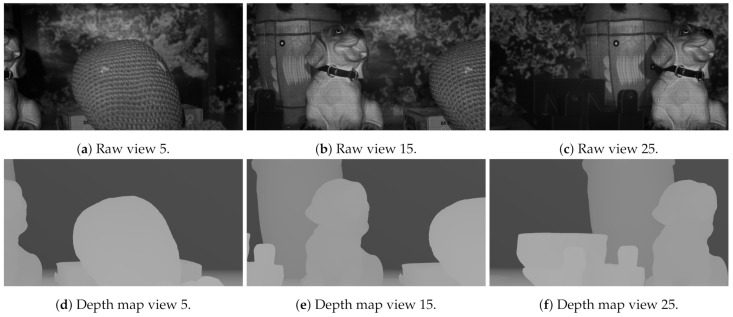
Raw images and depth maps from the HS multiview dataset generated.

This data set consists of a collection of real objects placed in front of the laboratory test acquisition system at different distances and with different textures. In this way, an HS multiview was generated using the HS camera moved across the linear actuator. After the acquisition, a 3D model ground truth was generated using a structured light 3D scanner, depicted in Figure 15. Then, using Blender (https://www.blender.org/, accessed on 10 June 2021), the camera array was simulated to acquire depth maps of the scene in the same positions as the real camera. Three raw images as well as their associated depth maps are included in Figure 16.

In this case, using the ground truth depth maps to measure the quality, there is no need to generate synthesized views. For this reason, GoRG depth maps are compared directly to ground truth depth maps using Root Mean Square Error (RMSE).

## 7. Results

In this section, first, the intermediate results that originated from the optimization stage of GoRG are presented. Then, GoRG is evaluated for YUV, showing the viability of generating high quality depth maps in operating room time constraints. Finally, the results for HS GoRG are presented. These results include objective quality, subjective quality, processing times, and stage profiling.

### 7.1. Development Stage Results

This section includes the experiments performed during the development stage of GoRG along with their results. It includes information about the kernels employed, the graph cuts filtering stage, and the interpolation analysis.

#### 7.1.1. Kernel Performance and Optimization

**Figure 17:**
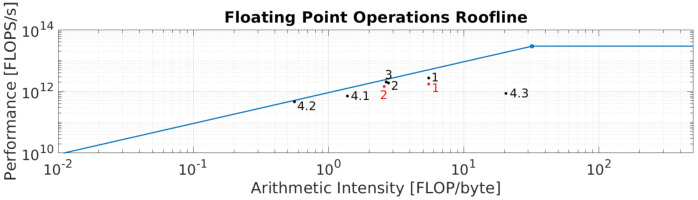
Roofline model with all of the kernels developed.

During Section 5, the kernels included in GoRG were introduced and explained. However, they followed a process of analysis and optimization that led to its current version. In this process, the roofline model played an important role, showing where the optimization was needed. For this reason, all of these kernels are explained, taking into consideration hardware performance indicators and introduced in a roofline chart. They are listed below:

1. Candidate index calculation (Algorithm 1): this kernel performs an independent matrix multiplication, scaling, and rounding for every spatial pixel and $Z_{candidate}$. This workload is mapped to the GPU with a grid where a thread address an image pixel and iterates then for the number of $Z_{candidates}$. In this way, Instruction Level Parallelism (ILP) [43] is increased, improving the performance due to a better GPU instruction pipelining. Compared to the same kernel addressing a thread for every pixel and $Z_{candidate}$, the increased ILP version achieves 1.76 instructions executed per cycle (Ipc) against 1.29 for the lower ILP version. With this change, a ×1.57 speed-up factor is obtained and limited by the memory storage of the indexes. This is depicted in the roofline chart in Figure 17 for point *1* in black (increased ILP) and red (lower ILP).
2. Cost calculation YUV (Algorithm 2): this kernel accumulates a L1 norm (subtraction and absolute value) in a 3×3 window for Y values and then another L1 norm for U and for V for every pixel and $Z_{candidate}$. This includes the load of 2×(3×3+1+1) values, their processing, and the storage of values. However, the values for the reference image are reused for all of the $Z_{candidates}$, which when combined with the increasingly discussed previous ILP discussion, leads to a grid structure where every thread addresses a pixel and iterates through $Z_{candidates}$. This allows for obtaining the load of the reference values before the loop, reducing the number of accesses. To do so, two approaches were tested: (i) the load of all of the reference values used by a thread in its private registers and (ii) the load of a shared memory window that includes all of the values in a wider region (32×32 tiles), using an overlapping block scheme. These tests revealed that shared memory for storing neighboring windows in tiles does not improve the performance of this kernel, as the levels of the L1 and L2 cache hit rate are very high for the register version, 88% and 33%, respectively. The register version is ×1.53 faster than the shared memory version. These kernels are depicted in the roofline chart in Figure 17 in point *2* in black (registers) and red (shared memory).
3. Cost calculation HS (Algorithm 3): this kernel is similar to the previous one, changing the L1 norm for the SIDSAM metric. Introducing this metric affects the computation needed as well as the number of load operations, which depends on the number of HS bands. Although SIDSAM adds logarithms, arc cosines, and square mean roots, which are computationally expensive for the GPU, this kernel is also memory bounded, needing access to a lot of data inefficiently. This is confirmed by a lower level of L1 and L2 cache hit rate, 40% and 21%, respectively. This kernel is shown in the roofline chart in Figure 17 in point *3*.
4. Smoothing/Reliability map calculation YUV (Algorithm 4), *Smoothing/Reliability map calculation HS (Algorithm 5)*, and *Motion map calculation YUV (Algorithm 6):* in these kernels, one thread is addressed for every spatial pixel, which processes their associated output map pixel. They represent a slight portion of the total processing time consumed by the application, less than 0.1 % (see Section 7); hence, the authors decided to not analyze them further. These kernels are depicted in Figure 17 as 4.1, 4.2, and 4.3, respectively.

#### 7.1.2. Graph Cuts Filtering

As introduced in Section 5.3, CUDA Cuts library is used within the alpha expansion method to refine the initial cube produced in the homography stage. This library may yield suboptimal results [34], producing small errors that are accumulated within the alpha expansion loop and originating larger errors in the final depth map. To overcome this problem, a filtering stage is included before and during alpha expansion loop.

As presented in Table 3 (only WS-PSNR is included for space reasons; IV-PSNR and VMAF do not add further information), this filtering introduces an increase of around 1.5% in quality and 9.2% in processing time, on average, for the YUV sequences tested, which is not considered a good trade-off. However, this average is strongly penalized by the sequence entitled PoznanFencing, with around a 30% time increase at the cost of only a 0.46% quality improvement. For all of the other sequences, filtering increases quality while increasing processing time by up to 6.5%, with only one case introducing losses. In addition, in two sequences, the time performed filtering is less than that without using this stage, meaning that the time including the process is amended by a faster convergence in graph cuts.

**Figure 18:**
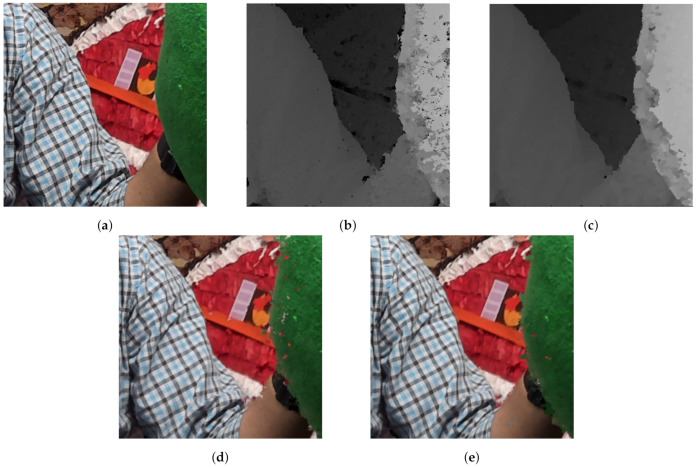
Detail of a sequence using GoRG with and without filtering. Original sequence Intel Frog, reproduced with permission from B. Salahieh (Intel). (**a**) Texture, (**b**) Depth map (no filtering), (**c**) Depth map (with filtering), (**d**) Synthesis of a virtual camera in the same position (no filtering) and (**e**) Synthesis of a virtual camera in the same position (with filtering).

In addition, the filtering subjective quality improvement is remarkable, as depth maps become smoother, which can be appreciated in Figure 18. This favors filling the holes that appear in the synthesis process.

#### 7.1.3. Interpolation Analysis

As introduced in Section 5.4, before implementing the interpolation stage in GoRG, a work of trade-off quality and processing times analysis and evaluation was performed. It is included in Table 4 (only WS-PSNR is included for space reason; IV-PSNR and VMAF do not add further information).

The results show that the quality differences between an interpolation with factor 1 and factor 8 are not significant for most of the sequences under test but are for only two sequences, with an increase higher to 1%. However, all of the sequences except OrangeKitchen have greater processing times, even reaching 30% increase. OrangeKitchen is a special case, where probably, the differences in the homography stage due to the interpolation caused a faster convergence in the graph cuts stage, leading to less processing time values.

These results show that the average quality increase between no interpolation and an interpolation of factor 8 is around 1.8%, whilst the average time processing increase is around 10.7% for the tested sequences. For this reason and prioritizing the acceleration, the authors decided not to include this stage in GoRG.

### 7.2. GoRG Evaluation Results with YUV Images

GoRG YUV quality results, which are averaged by view and frames, can be seen in Table 5. They are compared to DERS in the same conditions to analyze what is the quality-time trade-off achieved by GoRG, considering DERS as the quality reference.

In this table, averages of −0.93 dB, −0.60 dB, and −1.96% in WS-PSNR, IV-PSNR, and VMAF, respectively, are observed when comparing DERS to GoRG. The individual difference for each sequence is also shown, where many sequences obtain almost the same results in both applications and a more pronounced quality decrease in others, although it does not follow the same tendency for all of the metrics considered.

**Figure 19:**
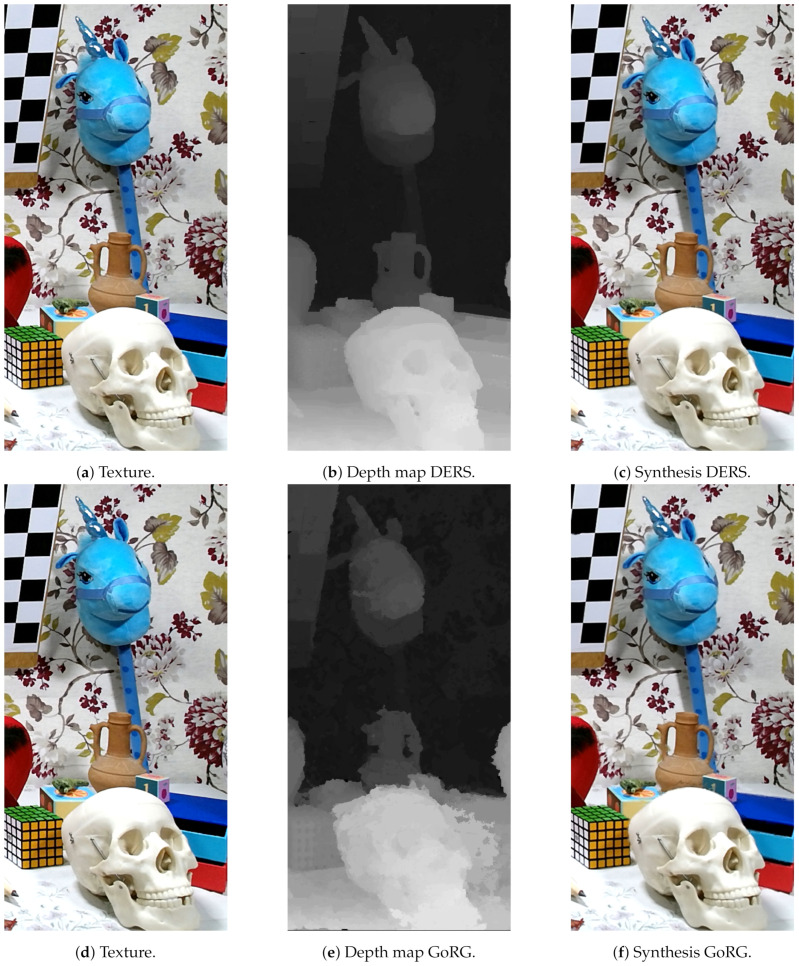
Detail of ULBUnicornA. Reproduced with permission from D. Bonatto et. al. (ULB-LISA), Electronic Imaging, 2020 [44].

**Figure 20:**
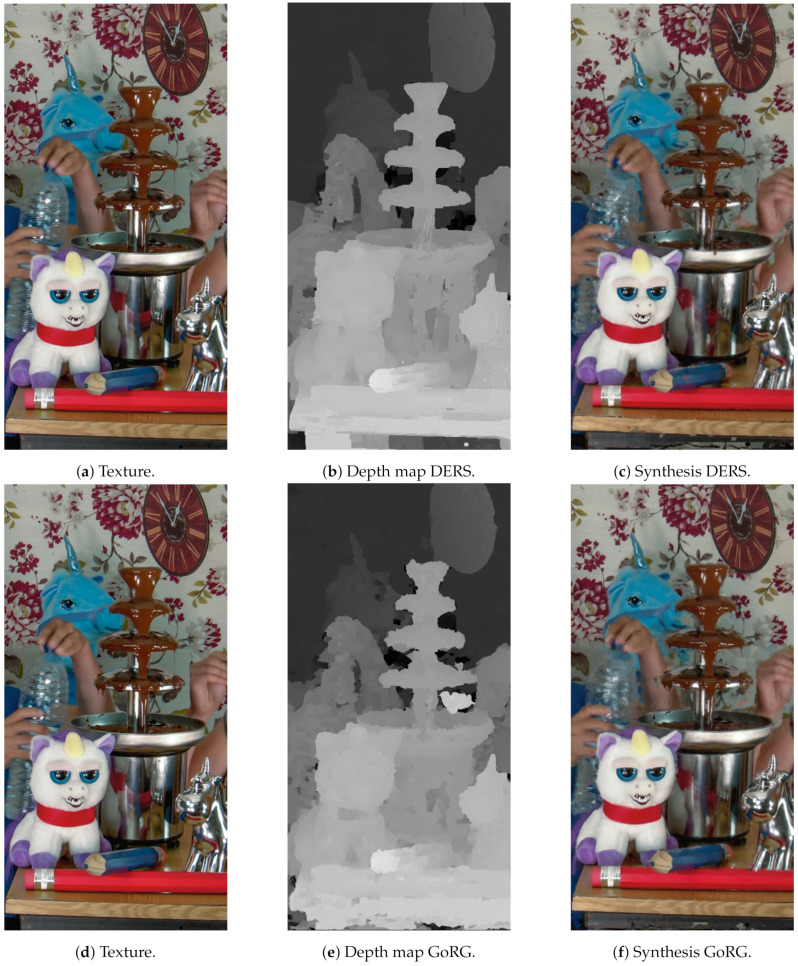
Detail of ULBBabyUnicorn. Reproduced with permission from D. Bonatto et. al. (ULB-LISA), Electronic Imaging, 2020 [44].

**Figure 21:**
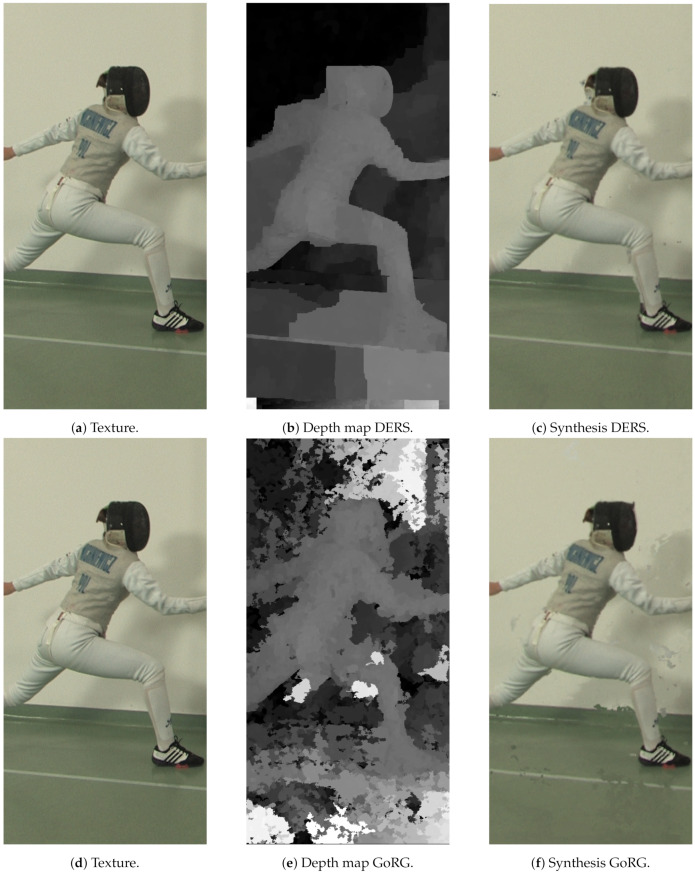
Detail of PoznanFencing. Reproduced with permission from O. Stankiewicz et al., IEEE Transactions on Multimedia, 2018 [45].

For subjective quality, Figure 19, Figure 20 and Figure 21 depict the results of several sequences for a certain view and frame. As can be seen, GoRG performs worse, especially for borders or areas with plain colors, although it is not especially noticeable for the synthesis images.

The processing time results are included in Table 6, comparing DERS to GoRG in terms of processing time per view and per frame and adding the speed-up. These results were obtained using, (i) for DERS, the Magerit-3 supercomputer of the Supercomputing and Visualization Center of Madrid (CeSViMa), with Intel Xeon Gold 6230 processors @ 2.10 GHz–3.90 GHz (20 cores), and (ii) for GoRG, a desktop computer with an Intel(R) Core(TM) i9-10900X CPU @ 3.70 GHz–4.50 GHz (20 cores) with an NVIDIA Geforce RTX 3090. In both cases, every view was processed by a single core of the CPU, however, in the case of GoRG, the CPU did not perform heavy-loaded computations, relying on the GPU for that purposes.

GoRG achieves an average processing time of 5.53 s, although it is completely dependent on the sequence content, as graph cuts is an iterative process and not every sequence has the same dimensions. Compared to DERS processing times, the average time is decreased from around hundred or thousand of seconds to less than 10 s, although some quality losses are introduced during the acceleration (detailed results are given in Table 6).

To conclude this section, the results of profiling for both applications are shown in Table 7. As can be seen, the time in DERS is mainly shared by the homography and graph cuts. In GoRG, the processing time consumed by graph cuts is more than the 98 %, being clearly the bottleneck. This occurs due to the difficulties found to parallelize graph cuts; an algorithm that needs to have all of the image pixels synchronized and hence hinders the possibility of treating them as independent threads in the GPU. For this reason, this algorithm needs to employ strategies where different parts of the image are computed serially, preventing the GPU from achieving good acceleration results. Hence, graph cuts becomes the performance bottleneck in GoRG.

### 7.3. GoRG Evaluation Results with HS Images

**Figure 22:**
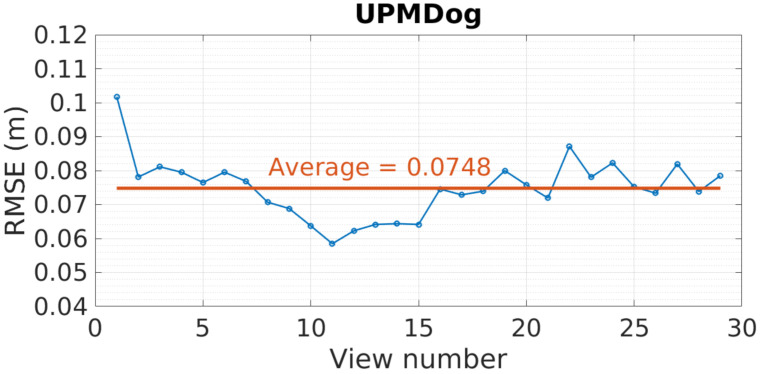
UPMDog view RMSE in meters.

GoRG HS quality results are introduced in Figure 22. These results are expressed using RMSE in meters for every view tested in the dataset. As can be seen, this error oscillates around the average value, which is near 7.5 cm and depends on the part of the scene captured. This error is not equally distributed within the image; for example, the depth surface of the octopus toy is estimated with an error of 0.5 cm, the dog is estimated with an error of 1.5 cm, and the drum is estimated with an error of 2 cm, while the background plain wall has an average error of 10 cm.

**Figure 23:**
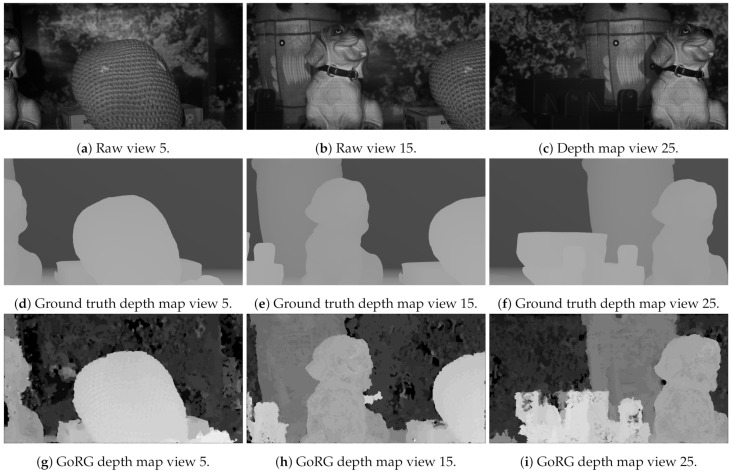
Results for UPMDog.

The subjective results can be observed in Figure 23, where the ground truth depth map is compared to GoRG results. These results show how GoRG depth maps are well estimated for the objects in the scene, also preserving borders. For non-textured objects such as background walls or the piece included in view 25, their spectral signature becomes difficult to differentiate, causing most of the errors in the depth estimations.

**Figure 24:**
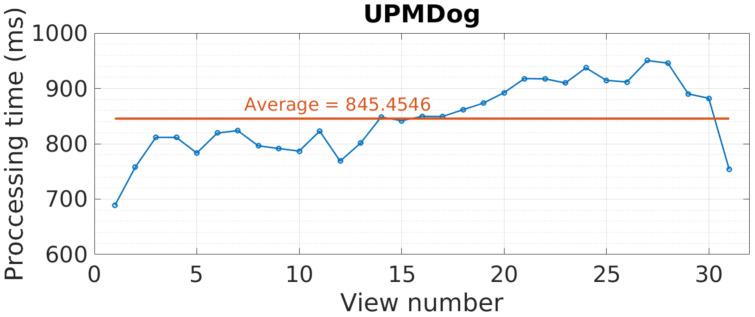
UPMDog view processing time in milliseconds.

The results for processing time can be seen in Figure 24. As before, processing time highly depends on the view number, oscillating around the average value, near 850 ms. This happens due to graph cuts, as its processing time depends on the image content. In addition, it is observed how these times are not as high as the ones in YUV, with the reason being the image spatial and depth resolution, although instead of three colors, these images have 25 bands.

To conclude this section, Table 8 shows the profiles for HS GoRG. As expected, the percentage of time is mainly shared between homography and graph cuts, with an increase of almost 20% in the homography compared to GoRG YUV. This is due to the fact that the homography needs to evaluate all of the spectral information for every pixel, increasing its computational load. In addition, graph cuts is processing smaller images, which reduces its complexity and processing time.

## 8. Conclusions

This works proposes an application-denominated Graph cuts Reference depth estimation in GPU (GoRG), a GPU-accelerated software intended for multiview high-quality depth estimation using HS images as input based on a SoA application named Depth Estimation Reference Software (DERS). GoRG is proposed to obtain high-detailed depth maps for HS images in brain tumor resection operations in real-time conditions. They are used to generate HS 3D models that improve both the classification of HS images and navigation by neurosurgeons.

In this work, DERS is analyzed and thoroughly explained stage by stage, serving as a foundation for GoRG. Its results for a varied set of sequences are shown and compared to GoRG, considered as a high-bound quality and processing time reference. In addition, this work includes stage profiling, showing that the processing time is shared between the homography and graph cuts stage.

Using DERS as the background, GoRG is explained in detail, focusing on its contributions: first, its GPU acceleration and, then, the use of HS images. For the former contribution, the acceleration strategies that followed are exposed and their implementation details are introduced. These implementations are then justified by giving the result of different experiments conducted to adjust and optimize the software. For the latter contribution, the approaches to using HS information in a depth estimation algorithm are introduced, using SIDSAM as a cost metric to obtain the initial depth estimation.

GoRG YUV, used as a prior step to the HS depth map generation, is compared to DERS, showing that the penalty in quality for using GoRG is, on average, −0.93 dB, −0.6 dB, and −1.96% for WS-PSNR, IV-PSNR, and VMAF, respectively. Subjective results exhibit differences mainly in borders and small objects for depth maps, although in synthesized images, they are difficult to differentiate. In terms of processing time, GoRG YUV achieves an average processing time of 5.53 s compared to around 13 min needed for DERS. These results showed that the real-time constraints defined for its application in the operating room were fulfilled with almost no quality losses, hence allowing for the development of an HS version accelerated with similar strategies.

GoRG HS was tested using a multiview HS scene emulating the operating room conditions: same illumination, reduced depth range, and close objects. This scene was acquired during this work along with a ground truth generated using a 3D scanner, enabling the exact characterization of GoRG HS. The results obtained show an RMSE error of 7.5 cm, on average, with most of the wrong pixels in the background, which can be appreciated in the HS test sequence. The processing time results show an average of around 850 ms for estimating a single view and frame, with differences depending on the image content. These results show its viability in real-time conditions, although the depth estimation quality is compromised. GoRG estimates low errors for the depth of objects near the scene, while farther objects are more poorly estimated. The worst results are found in the farthest plane of the scene. Since patient brains are expected to be close to the camera lens during a resection operation, the estimation error is likely to be low. Nevertheless, the system will be tested with real HS brain cubes to accurately find the achieved quality.

## 9. Future Lines

This work opens several future lines that will be addressed in the future. The first one focuses on its use and validation in the operating room, using brain tumor multiview HS images as input. The second one pursues the improvement of the software in terms of quality and time. As explained, the main bottleneck for both quality and processing time is the graph cuts process, hence developing a new GPU graph cuts library that does not introduce errors and is faster would severely improve the software functioning. In addition, new HS metrics will be analyzed and tested to contribute to the quality enhancement.

## Figures and Tables

**Table 1:** YUV sequences considered.

Sequences	Camera Configuration	Frames	Resolution	Depth Range (m)	Depth Steps
IntelFrog	linear 14×1	17	1920 × 1080	0.4–2	255
OrangeDancing	arc 14×3	17	1920 × 1080	1.2–14.2	255
OrangeKitchen	array 5×5	17	1920 × 1080	2.24–7.17	255
OrangeShaman	array 5×5	17	1920 × 1080	0.4–5.2	255
PoznanFencing2	arc 10×1	17	1920 × 1080	3.5–7	255
TechnicolorPainter	array 4×4	17	2048 × 1088	1.8–4.4	255
ULBBabyUnicorn3	array 5×3	17	1856 × 1032	1.5–4.2	255
ULBUnicornA	array 5×5	1	1920 × 1080	0.65–1.55	255

**Table 2:** HS sequence considered.

Sequences	Camera Configuration	Frames	Resolution	Depth Range (m)	Depth Steps
**UPMDog**	linear 1×30	1	832×448×25	0.5–1.1	100

**Table 3:** Filtering and no filtering results for YUV.

Sequence	WS-PSNR (dB)			Time (s)		
	No Filter	Filter	Diff	No Filter	Filter	Diff
OrangeShaman	37.16	37.75	1.56%	67.14	71.75	6.42%
OrangeDancing	31.68	32.51	2.56%	66.08	65.75	−0.50%
OrangeKitchen	30.21	30.09	−0.38%	97.60	104.96	7.02%
TechnicolorPainter	34.37	34.45	0.24%	122.64	129.08	4.99%
IntelFrog	26.82	27.88	3.83%	103.20	101.68	−1.49%
ULBUnicornA	26.62	27.37	2.73%	7.56	7.79	3.07%
ULBBabyUnicorn	26.80	27.24	1.61%	103.83	110.79	6.29%
PoznanFencing	25.76	25.87	0.46%	114.90	160.15	28.26%
**Average**	**29.93**	**30.40**	**1.55%**	**85.37**	**94.00**	**9.18%**

**Table 4:** Interpolation analysis in DERS.

Sequence	WS-PSNR (dB)			Time (s)		
	Interp 1	Interp 8	Diff	Interp 1	Interp 8	Diff
hlOrangeShaman	38.94	39.12	0.46%	14,057.43	14,265.91	1.48%
OrangeDancing	31.75	32.84	3.44%	5762.27	6986.97	21.25%
OrangeKitchen	31.67	31.94	0.85%	8908.57	8428.71	−5.39%
TechnicolorPainter	34.93	35.30	1.05%	12,603.97	15,352.62	21.81%
IntelFrog	28.05	28.03	−0.05%	16,382.00	21,381.65	30.52%
ULBUnicornA	25.13	27.34	8.81%	1178.70	1220.68	3.56%
ULBBabyUnicorn	27.64	27.82	0.66%	14,706.61	15,445.72	5.03%
PoznanFencing	28.16	28.20	0.13%	23,577.78	24,450.09	3.70%
**Average**	**30.78**	**31.32**	**1.92%**	**12,147.17**	**13,441.54**	**10.25%**

**Table 5:** Objective results for DERS and GoRG.

Sequence	WS-PSNR (dB)			IV-PSNR (dB)			VMAF (%)		
	DERS	GoRG	Diff	DERS	GoRG	Diff	DERS	GoRG	Diff
OrangeShaman	39.12	37.75	−1.37	44.13	43.80	−0.33	82.20	80.81	−1.99
OrangeDancing	32.84	32.51	-0.33	42.05	41.76	−0.28	78.65	77.94	−0.72
OrangeKitchen	31.94	30.09	−1.85	40.13	38.48	−1.65	81.72	77.38	−4.34
TechnicolorPainter	35.30	34.45	−0.85	43.43	43.37	−0.06	85.81	81.69	−4.11
IntelFrog	28.03	27.88	−0.15	37.98	37.77	−0.21	73.40	75.29	1.89
ULBUnicornA	27.34	27.37	0.03	37.62	37.46	−0.16	79.38	79.56	0.18
ULBBabyUnicorn	27.82	27.24	−0.58	35.20	34.44	−0.75	66.86	67.02	0.16
PoznanFencing	28.20	25.87	−2.33	36.64	35.28	−1.37	59.56	52.81	−6.75
**Average**	31.32	30.40	−0.93	39.65	39.05	−0.60	76.02	74.06	−1.96

**Table 6:** Average processing time per camera and per frame for DERS and YUV-GoRG (average time needed to process a depth map frame of one camera).

Sequence	Processing Time (s)		
	DERS	GoRG	Factor
**OrangeShaman**	839.17	4.22	199
**OrangeDancing**	411.00	3.87	106
**OrangeKitchen**	495.81	6.17	80
**TechnicolorPainter**	903.10	7.59	119
**IntelFrog**	1257.74	5.98	210
**ULBUnicornA**	1220.68	7.79	157
**ULBBabyUnicorn**	908.57	6.52	139
**PoznanFencing**	1438.24	9.42	153
**Average**	790.68	5.53	143

**Table 7:** Profiling per stages for DERS and GoRG.

Application	Read Cameras & Interpolation	Auxiliary Maps	Homography	Graph Cuts
DERS	5.05% (39.5 s)	0.25 % (2 s)	53.38% (419 s)	41.34 % (324 s)
GoRG	0.17 % (9 ms)	0.01 % (0.6 ms)	0.85 % (57 ms)	98.70 % (5.3 s)

**Table 8:** Profiling per stages for HS GoRG.

Read Cameras	Auxliary Maps	Homography	Graph Cuts
5.73% (48.45 ms)	0.09% (0.765 ms)	19.39% (164.9 ms)	74.79% (637.5 ms)

## Data Availability

The data presented in this study are available on request from the corresponding author. The data are not publicly available due to privacy reasons.
